# Glycoprotein NMB: a novel Alzheimer’s disease associated marker expressed in a subset of activated microglia

**DOI:** 10.1186/s40478-018-0612-3

**Published:** 2018-10-19

**Authors:** Melanie Hüttenrauch, Isabella Ogorek, Hans Klafki, Markus Otto, Christine Stadelmann, Sascha Weggen, Jens Wiltfang, Oliver Wirths

**Affiliations:** 1 Department of Psychiatry and Psychotherapy, University Medical Center (UMG), Georg-August-University, Von-Siebold-Str. 5, 37075 Göttingen, Germany; 2 Department of Neuropathology, Heinrich-Heine-University, Düsseldorf, Germany; 3 Department of Neurology, University of Ulm, Ulm, Germany; 4 Department of Neuropathology, University Medical Center, Georg-August-University, Göttingen, Germany; 5 German Center for Neurodegenerative Diseases (DZNE), Göttingen, Germany

**Keywords:** Alzheimer’s disease (AD), Glycoprotein nonmetastatic melanoma protein B (GPNMB), Neuroinflammation, Activated microglia, Sporadic AD patients, Transgenic mice, 5XFAD

## Abstract

Alzheimer’s disease (AD) is an irreversible, devastating neurodegenerative brain disorder characterized by the loss of neurons and subsequent cognitive decline. Despite considerable progress in the understanding of the pathophysiology of AD, the precise molecular mechanisms that cause the disease remain elusive. By now, there is ample evidence that activated microglia have a critical role in the initiation and progression of AD. The present study describes the identification of Glycoprotein nonmetastatic melanoma protein B (GPNMB) as a novel AD-related factor in both transgenic mice and sporadic AD patients by expression profiling, immunohistochemistry and ELISA measurements. We show that GPNMB levels increase in an age-dependent manner in transgenic AD models showing profound cerebral neuron loss and demonstrate that GPNMB co-localizes with a distinct population of IBA1-positive microglia cells that cluster around amyloid plaques. Our data further indicate that GPNMB is part of a microglia activation state that is only present under neurodegenerative conditions and that is characterized by the up-regulation of a subset of genes including *TREM2*, *APOE* and *CST7*. In agreement, we provide in vitro evidence that soluble Aβ has a direct effect on *GPNMB* expression in an immortalized microglia cell line. Importantly, we show for the first time that GPNMB is elevated in brain samples and cerebrospinal fluid (CSF) of sporadic AD patients when compared to non-demented controls.

The current findings indicate that GPNMB represents a novel disease-associated marker that appears to play a role in the neuroinflammatory response of AD.

**Electronic supplementary material:**

The online version of this article (10.1186/s40478-018-0612-3) contains supplementary material, which is available to authorized users.

## Introduction

Alzheimer’s disease (AD) is a progressive, age-associated neurodegenerative disorder and the most frequent cause of dementia among the elderly population. Major neuropathological hallmarks of AD include an abnormal accumulation of extracellular β-amyloid (Aβ) peptides and intraneuronal neurofibrillary tangles composed of hyperphosphorylated tau protein. The disease involves extensive loss of synapses and neuronal death in the cerebral cortex and hippocampus, leading to gradual memory loss and cognitive decline. Despite considerable efforts during the last decades to find an efficacious therapy to halt or reverse AD pathology, currently available drugs allow at best an alleviation of the symptoms but do not affect the underlying cause of the disease [44].

Apart from amyloid plaques and intracellular tau aggregates, neuroinflammation represents an additional hallmark of AD. An increase in neuroinflammatory markers such as nitric oxide, interleukin-1β (IL-1β) and tumor necrosis factor (TNF-α) has been widely reported in brains of both Alzheimer’s disease patients and transgenic AD models (reviewed in [14]). Emerging evidence suggests that instead of solely being a passive response to aberrant protein aggregation in the brain, persistent neuroinflammation might play a causal role in the pathogenesis of AD. This hypothesis is supported by recent genome-wide association studies (GWAS) linking specific polymorphisms in inflammation-associated genes such as complement receptor-1 (*CR1*) [26], CD33 [13, 31] or triggering receptor expressed on myeloid cells-2 (*TREM2*) [8] to an increased risk for AD. Therefore, a detailed understanding of immunological processes associated with the disease has become a major goal in Alzheimer’s research in order to evaluate modulation of neuroinflammation as a new therapeutic modality.

In a previous project, we performed a whole-brain transcriptome study to identify genes differentially expressed in the brains of 6-month-old APP/PS1KI mice compared to age-matched PS1KI and WT controls [51]. APP/PS1KI mice are a widely used AD model showing profound neuron loss in several brain regions, as well as working memory deficits and disturbed long-term potentiation [3, 5, 55]. The majority of genes that we discovered to be upregulated in APP/PS1KI mice compared to both control groups were implicated in inflammation-associated pathways and included intensively studied genes such as *TREM2*. Intriguingly, one of the most strongly up-regulated genes in the APP/PS1KI model was Glycoprotein nonmetastatic melanoma protein B (*GPNMB*), a gene that so far has not been implicated in AD [51].

GPNMB (also known as osteoactivin, OA) is a type I transmembrane glycoprotein that was initially described in a poorly metastatic melanoma cell line [52]. GPNMB is at least partially localized to the cell surface and ectodomain shedding by ADAM10 can release its large N-domain into the extracellular space [40]. Since its identification, GPNMB expression has been detected in multiple tissues such as bone, kidney and skeletal muscle where it is implicated in various cellular processes like cell differentiation, tumor progression and tissue regeneration [1, 25, 34, 53]. Furthermore, there is profound evidence that GPNMB has a function as a negative regulator of inflammatory processes. In macrophages, overexpression of GPNMB reduced the secretion of proinflammatory cytokines in vitro [39]. More recent data (in peripheral tissues) further indicate that GPNMB promotes the polarization of macrophages into an anti-inflammatory “M2” status, which results in the secretion of anti-inflammatory cytokines such as IL-10 and TGF-β [59, 61]. Interestingly, in the central nervous system, GPNMB expression was identified within motor neurons, radial glia and most abundantly in microglia cells, which are the resident immune cells of the brain. Therefore, it has been proposed that GPNMB might also play a role in inflammatory processes in the CNS [15]. Furthermore, GPNMB has been shown to be elevated in brain and/or plasma of numerous neurodegenerative diseases such as Gaucher disease [23, 62], Niemann-Pick Type C disease [28] and amyotrophic lateral sclerosis (ALS) [48]. However, the impact of GPNMB overexpression on the pathophysiology of these diseases has not been elucidated.

The aim of the present work was to investigate a potential role of GPNMB in transgenic AD mouse models and human patients with sporadic AD. We demonstrate an age-dependent increase in *GPNMB* mRNA and protein levels in different AD mouse models. In addition, we discovered that GPNMB expression increases in parallel with Aβ plaque deposition, therefore reflecting disease severity. Moreover, increased GPNMB levels were observed in the cerebrospinal fluid (CSF) and brains of human patients with sporadic AD.

## Material and methods

### Transgenic mice

The generation of 5XFAD mice (Tg6799) has been described previously [33]. In brief, 5XFAD mice overexpress APP695 carrying the Swedish, Florida and London mutations under the control of the murine Thy-1 promoter. Additionally, human presenilin-1 (PSEN1), carrying the familial Alzheimer’s disease (FAD)-linked mutations M146 L and L286 V, is also expressed under the control of the murine Thy-1 promoter. 5XFAD mice used in this study were backcrossed for more than 10 generations to C57Bl/6 J wild-type mice (WT) from the Jackson Laboratory (Jackson Laboratories, Bar Harbor, ME, USA) to obtain an incipient congenic line on a C57BL/6 J genetic background.

The generation of APP/PS1KI mice has also been described [5]. APP/PS1KI mice express human mutant APP751 carrying the Swedish and London mutations under the control of the murine Thy-1 promoter. In addition, murine PSEN1 containing the M233 T and L235P mutations is expressed under the control of the endogenous mouse PSEN1 promoter. The APP/PS1KI mouse model was a generous gift of Dr. Laurent Pradier, Sanofi-Aventis, Paris, France.

The APP23 model was originally described by Sturchler-Pierrat and colleagues [46]. In this AD mouse model, human APP751 with the Swedish double-point mutation K670 M/N671 L is overexpressed under the control of the murine Thy-1 promoter. APP23 mice were a generous gift of Dr. Mathias Staufenbiel, Novartis, Basel, Switzerland. All animals were handled according to German guidelines for animal care.

### Patient samples

#### Human brain samples

Human frozen brain samples from sporadic AD (*n* = 9, mean age 79.78 ± 11.28 years, Braak stage V-VI) and non-demented control subjects (NDC, *n* = 9, mean age 82 ± 9.77 years, Braak stage I-II), as well as paraffin-embedded AD and NDC samples for immunohistochemistry were obtained from the Netherlands Brain Bank. The present study was approved by the ethics committee of the University Medical Center Göttingen (12/1/15). Details regarding autopsy procedure can be found at www.brainbank.nl. Characteristics of the study cohort are presented in Additional file 1.

#### Human CSF samples

Human cerebrospinal fluid (CSF) and corresponding serum samples from patients suffering from sporadic AD (*n* = 10, mean age 70.4 ± 7.56 years) and NDC subjects (*n* = 10, mean age 62.5 ± 9.32) were obtained by lumbar puncture, centrifuged, aliquoted and stored within 2 h at − 80 °C until analysis. All patients were seen at the Department of Neurology in Ulm (Ethical approval number 20/10). Characteristics of the study cohort are presented in Additional file 1.

### Cell culture and treatment conditions

Murine immortalized microglial BV-2 cells were grown in DMEM/F-12 media supplemented with 10% heat-inactivated fetal bovine serum (FBS, Biochrom), 2 mM L-Alanyl-L-Glutamine (Sigma-Aldrich) and non-essential amino acids (Sigma-Aldrich). SH-SY5Y cells stably overexpressing APP695 containing the Swedish mutation K670 N/M671 L and carrying a Myc tag and a carboxy-terminal Flag tag [29] were cultured in DMEM/F-12 media supplemented with 10% heat-inactivated FBS, 2 mM L-Alanyl-L-Glutamine, non-essential amino acids, and 50 μg/ml Hygromycin B (Carl Roth). Cells were maintained at 37 °C in a humidified atmosphere containing 5% CO_2_. BV-2 cells were seeded into 9.6 cm^2^ petri dishes (3 × 10^5^ cells/dish; *n* = 6/treatment group), cultured for 24 h, and then stimulated with lipopolysaccharide (LPS) (0.1 μg/ml), conditioned media of SH-SY5Y cells, or Aβ_1–42_ (5 μM) for 24 h at 37 °C with 5% CO_2_. Then cells were collected for RNA extraction. Aβ_1–42_ peptides were purchased from Peptide Speciality Laboratory (PSL) and resuspended in 20 mM NaOH (final concentration, 1 mg/ml) directly before treatment of BV-2 cells. To obtain Aβ-containing conditioned SH-SY5Y media, cells were plated in a 175cm^2^ cell culture flask w/o Hygromycin B and incubated for 48 h. The conditioned media were collected, centrifuged for 5 min at 1400 rpm, and applied to BV-2 cells for the indicated period of time.

### Immunohistochemistry and immunofluorescence analyses

Mice were killed by CO_2_ anesthetization followed by cervical dislocation. Brains and spinal cords were carefully dissected and post fixation for at least one week was carried out in 4% phosphate-buffered formalin at 4 °C before the tissue was embedded in paraffin. Immunohistochemistry was performed on 4 μm paraffin sections as described previously [38]. The following antibodies were used: anti-Aβ antibody 24311 (1:500, [41]), IBA1 (1:500; #234004, Synaptic Systems) and GPNMB (1:500, Santa Cruz). Biotinylated secondary anti-rabbit, anti-guinea pig and anti-goat antibodies (1:200) were purchased from Dako or Jackson Immunoresearch. The staining was visualized using the avidin-biotin complex method with a VECTASTAIN kit (Vector Laboratories) and diaminobenzidine (DAB) as a chromogen providing a reddish-brown color with hematoxylin as nuclear counterstain.

For double-immunofluorescence staining, polyclonal goat anti-GPNMB antibody (1:500, AF2330, R&D Systems) was combined with IC16 (1:1000, against the N-terminus of Aβ), GFAP (1:500, #173004, Synaptic Systems), NeuN (1:300, MAB377, Millipore) and IBA1 (1:500, #234004, Synaptic Systems), respectively. The staining was visualized using Alexa Fluor 594- and Alexa Fluor 488-conjugated secondary antibodies (1:750, Jackson Immunoresearch) and analyzed using a Nikon Eclipse Ti-E fluorescent microscope.

### Elisa

GPNMB levels were measured in human and mouse brain tissue, mouse spinal cord tissue and in human CSF and serum samples using commercially available mouse (DY2330) or human Osteoactivin/GPNMB ELISA (DY2550) sets according to the manufacturer’s instructions (R&D systems, Abingdon, UK).

Proteins were extracted from human and mouse brain samples as well as from mouse spinal cord samples. Frozen tissue was weighed and homogenized in 0.7 ml Tris-buffered saline (TBS) buffer (120 mM NaCl, 50 mM Tris, pH 8.0 supplemented with complete protease inhibitor cocktail (Roche Diagnostics, Indianapolis, IN, USA)), per 100 mg tissue by using a Dounce homogenizer (800 rpm). The resulting homogenate was centrifuged at 17,000 x *g* for 20 min at 4 °C. The supernatant containing TBS-soluble proteins was stored at − 80 °C. The pellet was dissolved in 2% sodium dodecyl sulfate (SDS) and sonicated followed by a centrifugation step at 17,000 x *g* for 20 min at 4 °C. The supernatant, which contained SDS-soluble proteins, was incubated with 1 μl of Benzonase under rotating conditions for 10 min at room temperature in order to reduce viscosity and stored at − 80 °C.

### Real-time PCR

For real-time RT-PCR analysis, WT, 5XFAD, APP/PS1KI and APP23 mice (*n* = 3–6 per group) or RNA extracts from BV2 cells (*n* = 6) were used. For RNA isolation, deep-frozen brain hemispheres or spinal cord tissue were homogenized in TriFast reagent (Peqlab) essentially as described previously [16]. Deep frozen liver samples were homogenized in 1 ml TriFast reagent (Peqlab) per 100 mg tissue using a glas-teflon homogenizer. BV2 cell pellets were homogenized manually in 1 ml TriFast reagent by repetitive pipetting. DNAse digestion and reverse transcription of the purified RNA samples were carried out according to the protocol of the manufacturer (Thermo Fisher). RT-PCR was performed using a Stratagene MX3000 Real-time Cycler. The SYBR green based FastStart Universal SYBR Green (Roche) containing ROX as an internal reference dye was used for amplification. Relative expression levels were calculated using the 2^- ∆∆Ct^ method and normalized to the housekeeping gene β-actin [43]. Primer sequences can be found in Additional file 2.

### Statistical analysis

Differences between groups were tested by either one-way analysis of variance followed by Tukey’s multiple comparisons test or unpaired *t*-tests. All data were expressed as mean ± SD. Significance levels are indicated as follows: ***p < 0.001; **p < 0.01; *p < 0.05. All calculations were performed using GraphPad Prism version 6.07 for Windows (GraphPad Software, La Jolla, CA, USA).

## Results

### *GPNMB* expression levels increase with disease progression in distinct transgenic AD mouse models

**Fig. 1:**
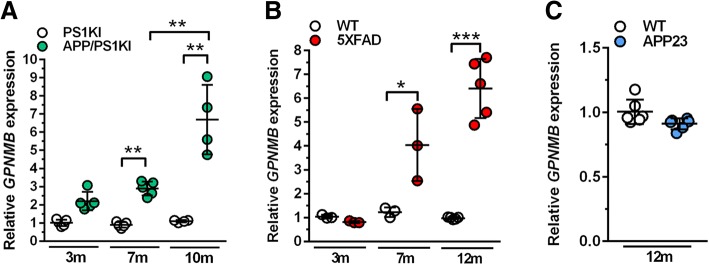
*GPNMB* mRNA expression increases in an age- and dose-dependent manner in some Alzheimer’s disease mouse models. (**a**) Cerebral *GPNMB* mRNA levels increased in an age-dependent manner in APP/PS1KI compared to PS1KI control mice. (**b**) In 5XFAD mice, levels of *GPNMB* mRNA started to be significantly increased at 7 months of age when compared to wild-type (WT) control mice. 12-month-old 5XFAD mice showed even higher *GPNMB* levels. (**c**) In 12-month-old APP23 mice, *GPNMB* gene expression levels were not increased compared to WT animals. All data are given as mean ± SD. ***P < 0.001; **P < 0.01; *P < 0.05

As previously reported, we initially found *GPNMB* mRNA levels to be significantly up-regulated in 6-month-old APP/PS1KI mice when compared to control mice in a whole-brain deep sequencing analysis [51]. In order to test whether *GPNMB* mRNA up-regulation occurs during normal aging or if *GPNMB* expression is regulated in a disease-state dependent manner, RNA of whole brain hemispheres from 3-, 7- and 10-month-old APP/PS1KI and PS1KI control mice was extracted and *GPNMB* expression levels were analyzed using RT-PCR. We observed a disease-state dependent upregulation of *GPNMB* mRNA levels in APP/PS1KI mice but not in PS1KI mice. In good agreement with our previous study, *GPNMB* mRNA expression was significantly increased in 7-month-old APP/PS1KI mice compared to controls (*p* < 0.01) [51]. At 12 months of age, *GPNMB* expression increased even further when compared to 7-month-old APP/PS1KI mice (*p* < 0.01; Fig. 1a).

To determine whether *GPNMB* mRNA up-regulation also occurs in other AD mouse models, potentially indicating a general event during AD pathology progression, RT-PCR analyses of brain hemispheres of 3-, 7- and 12-month-old 5XFAD and age-matched WT control animals were performed. While *GPNMB* expression was unchanged in 3-month-old 5XFAD mice when compared to WT animals, mRNA levels were significantly upregulated at 7 months of age (*p* < 0.05). At 12 months of age, *GPNMB* mRNA levels in 5XFAD mice were even further increased compared to WT mice (*p* < 0.001), where no age-dependent changes were detectable (Fig. 1b). Interestingly, in APP23 mice, another frequently studied mouse model of AD, no *GPNMB* up-regulation was detected in 12-month-old APP23 mice as compared to WT control animals (Fig. 1c).

### Cellular localization and distribution of GPNMB in the CNS of AD mouse models

**Fig. 2:**
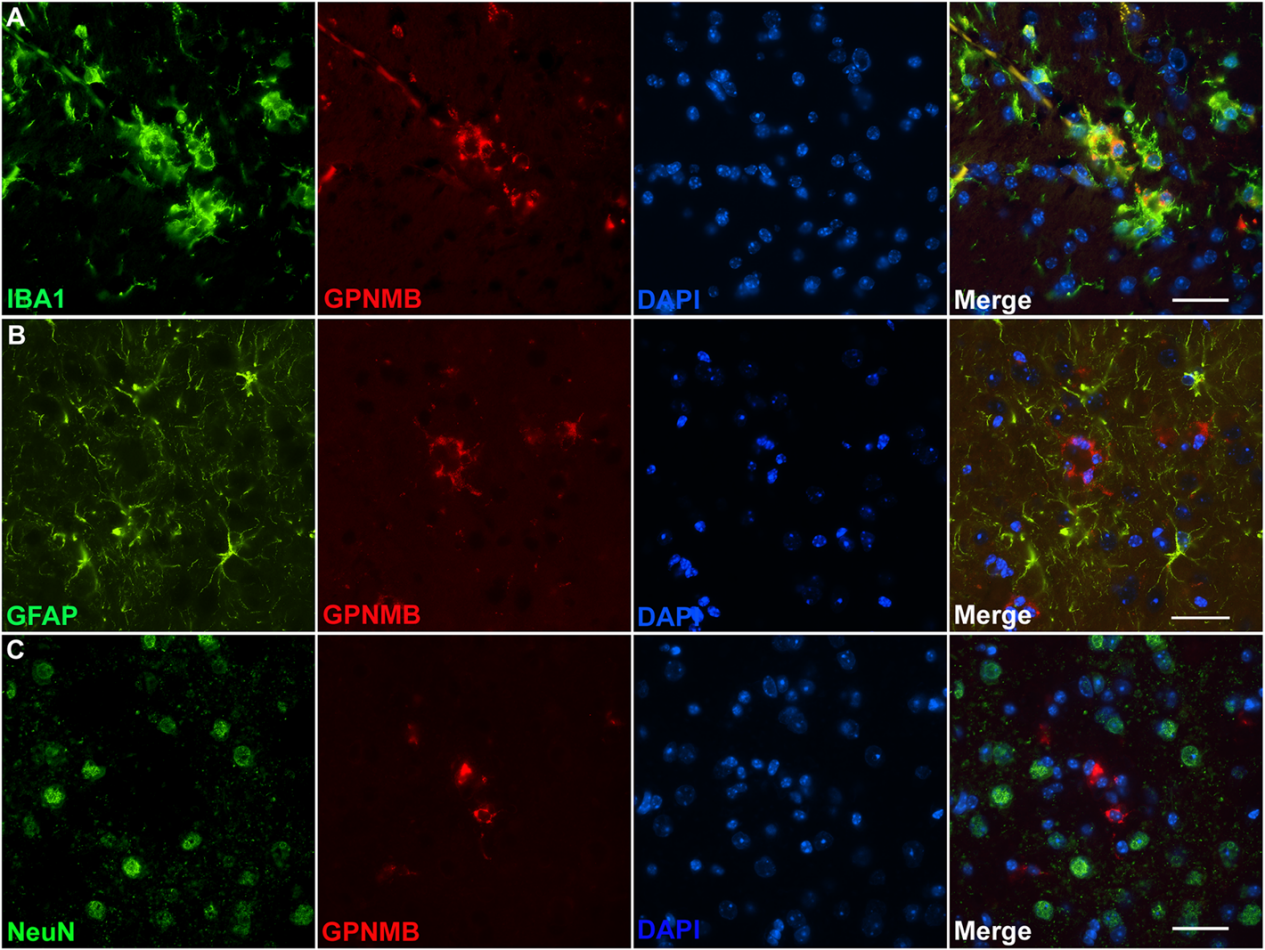
GPNMB co-localizes with IBA1-positive microglia cells in 5XFAD brains. (**a-c**) Double immunostaining with antibodies against GPNMB and IBA1 revealed a cellular co-localization in 12-month-old 5XFAD brains, while no co-localization was seen with the astrocytic marker GFAP or the neuronal marker NeuN. Scale bar: A-C = 33 μm

Next, we aimed to investigate the cellular localization of GPNMB using double staining with cellular marker proteins such as NeuN for neurons, GFAP for astrocytes and IBA1 as a marker for microglia and macrophages. While no co-localization of GPNMB was observed with NeuN or GFAP, abundant GPNMB immunoreactivity could be detected in cells positive for the microglia/macrophage marker IBA1 in 5XFAD mice (Fig. 2).

**Fig. 3:**
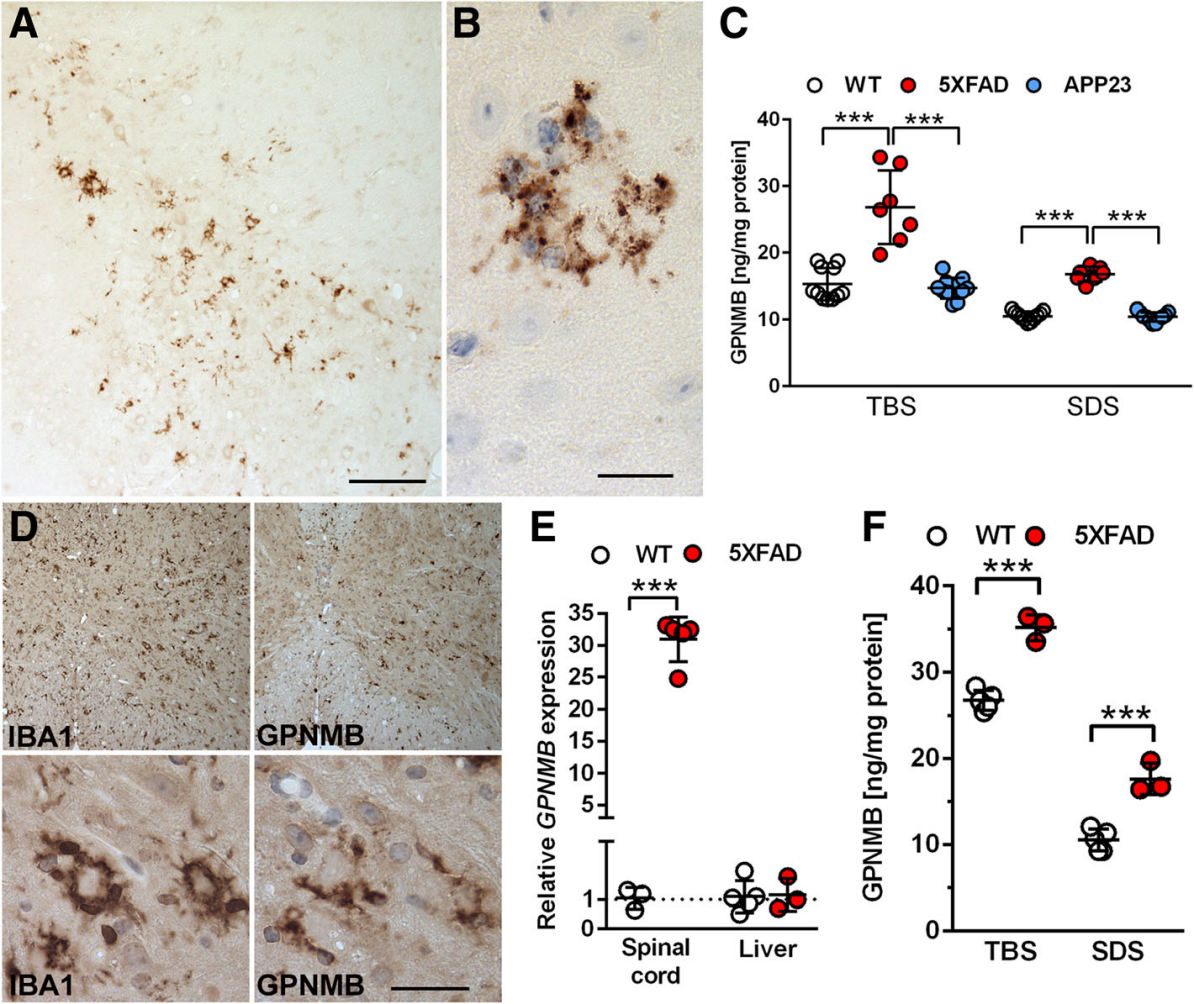
Increased GPNMB protein levels and cerebral expression pattern in 5XFAD mice. (**a**) Abundant GPNMB staining was detected throughout the whole brain in 12-month-old 5XFAD mice, e.g. in the subiculum. (**b**) High-power view of GPNMB-positive cells surrounding an amyloid plaque core. (**c**) Quantitative analysis of GPNMB protein levels in TBS- and SDS-soluble brain fractions of 12-month-old WT, 5XFAD and APP23 mice revealed a highly significant increase in GPNMB levels in 5XFAD mice compared to WT animals, while no elevated levels could be observed in APP23 mice. **(d)** GPNMB immunoreactivity was also demonstrated in the spinal cord (SC) of 12-month-old 5XFAD mice. GPNMB was abundantly expressed throughout the whole SC tissue and showed co-localization with IBA1-positive microglia cells (parallel sections). (**e**) As demonstrated by RT-PCR analyses, 5XFAD mice showed a highly significant increase in *GPNMB* mRNA levels in the SC when compared to WT controls. However, no such differences were observed in peripheral liver samples serving as a non-neuronal control tissue. Enzyme-linked immunosorbent assay revealed highly increased GPNMB protein level in 5XFAD SC tissue compared to WT controls (**f**). All data are given as mean ± SD. ***P < 0.001. Scale bar: A = 133 μm, B = 25 μm; D = 200 μm (upper panel), 20 μm (lower panel)

In order to better characterize the localization of GPNMB in the CNS of AD transgenic mice, further immunohistochemical stainings were performed in brain and spinal cord of 12-month-old 5XFAD mice compared to age-matched WT controls. In 5XFAD mice, GPNMB immunoreactivity was observed throughout the whole brain with particular abundance in regions known for high Aβ plaque load in this model, such as subiculum (Fig. 3a, b), cortex or thalamus, while no signal could be detected in APP23 or WT control mice (Additional files 3 , 4). 5XFAD mice start to develop amyloid pathology as early as two months of age in the subiculum and deep cortical layers. At 12 months of age, the model shows a massive Aβ plaque load in various brain regions, which is accompanied by extensive astro- and microgliosis [19, 33]. To investigate whether GPNMB accumulates in parallel with amyloid plaque deposition in this model, GPNMB and Aβ immunoreactivity were quantified in 2.5-, 7- and 12-month-old 5XFAD mice in the cortex, subiculum, dentate gyrus and thalamus. Compared to 2.5-month-old mice, aged mice (7 m and 12 m, respectively) revealed a significant increase in GPNMB levels in all regions analyzed. The same was true for extracellular amyloid deposition as demonstrated with antibody 24311 detecting a variety of different Aβ isoforms (Additional file 3). Hence, cerebral GPNMB accumulation increases in an age-dependent manner in 5XFAD mice, resembling β-amyloid accumulation.

In order to more accurately quantify whether the increased *GPNMB* mRNA expression in the brains of 5XFAD mice correlated with an elevation in GPNMB protein levels, a sandwich ELISA was used to measure GPNMB levels in TBS-soluble and SDS-soluble brain fractions from both 5XFAD and APP23 mice. Analysis of 12-month-old 5XFAD mice revealed a highly significant elevation of GPNMB protein levels when compared to age-matched WT or APP23 mice (*p* < 0.001). A similar pattern was seen in the SDS-soluble fraction, showing significantly increased GPNMB protein levels in 12-month-old 5XFAD mice (*p* < 0.001) when compared to age-matched WT or APP23 animals, respectively (Fig. 3c). These measurements also confirmed that GPNMB protein levels were not increased in 12-month-old APP23 mice as compared to WT control animals.

As GPNMB has been previously implicated in motor neuron diseases [4], spinal cord samples from aged 5XFAD mice were analyzed where Aβ pathology has been previously demonstrated [19]. As seen in the brain, abundant GPNMB-immunoreactivity was observed throughout the whole spinal cord, largely resembling the IBA1 staining profile (Fig. 3d). Furthermore, a comparison of *GPNMB* mRNA expression levels in the spinal cord revealed a highly significant increase in 5XFAD compared to age-matched WT mice (*p* < 0.001). Liver samples were used as a control peripheral tissue and did not show any induction of GPNMB levels in aged 5XFAD mice (Fig. 3e). Likewise, protein extracts from 5XFAD and age-matched WT spinal cord samples revealed a significant elevation of GPNMB protein levels in both TBS- and SDS-soluble spinal cord fractions of 5XFAD mice (*p* < 0.001, respectively) (Fig. 3f). For a subset of analyzed animals, both RNA and protein samples from the same mouse was available. Statistical analysis revealed a high correlation for GPNMB RNA and protein levels (Additional file 5).

**Fig. 4:**
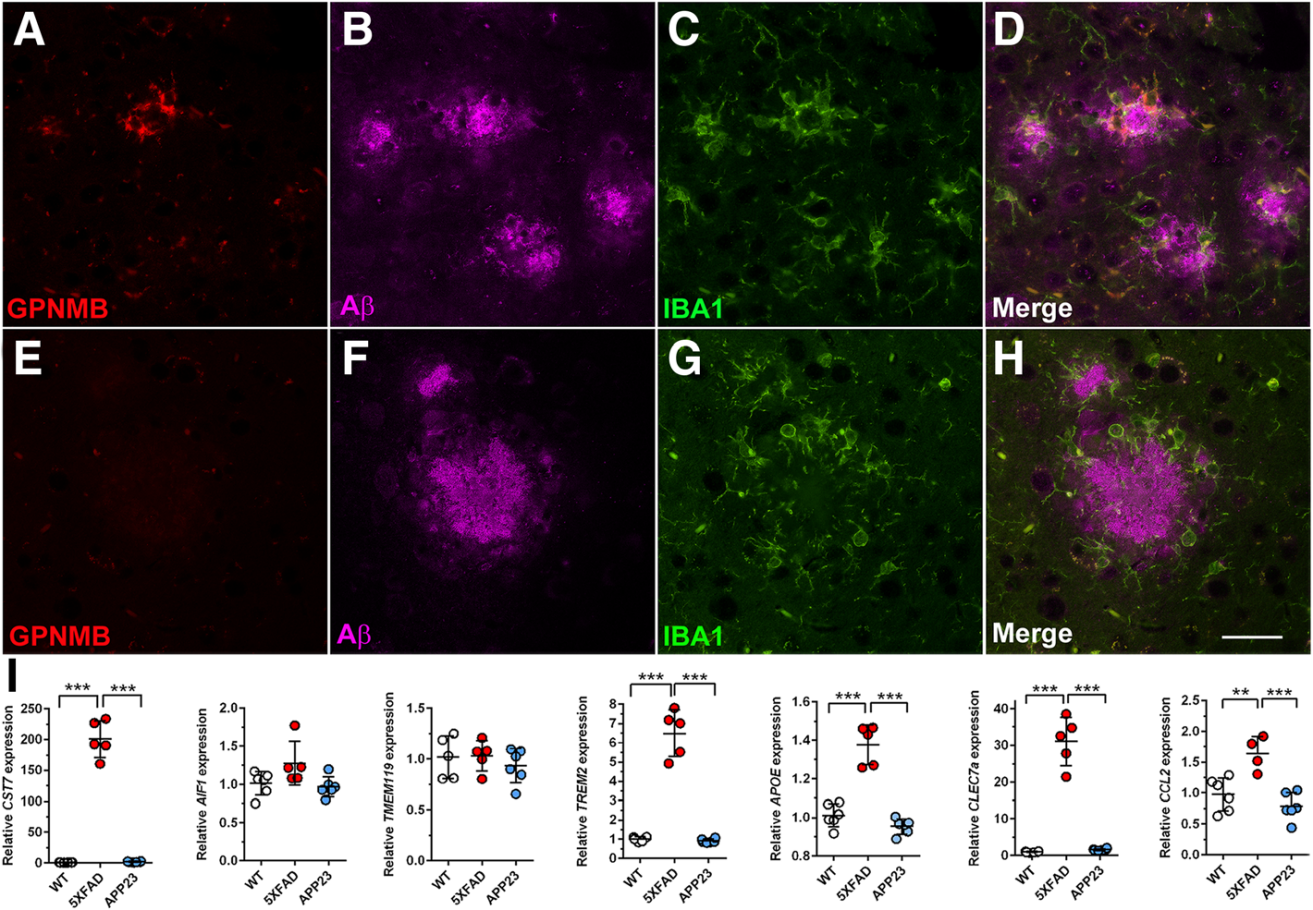
GPNMB/IBA-1-positive microglia cells cluster around individual plaque cores in 5XFAD brains. Triple immunofluorescence staining using antibodies against GPNMB (**a**), Aβ (**b**) and IBA1 (**c**) demonstrated the spatial co-localization of GPNMB-positive microglia cells around amyloid plaque cores in 12-month-old 5XFAD brains (**d**). Even though APP23 mice showed numerous activated microglia cells (**g**) clustered around amyloid plaques (**f**), no GPNMB signal could be detected (**e**,**h**). (**i**) RT-PCR analyses revealed significantly increased mRNA levels of *CST7*, *TREM2*, *APOE*, *CLEC7A* and *CCL2* in 5XFAD brains when compared to WT and APP23 mice. However, levels of *AIF1* and *TMEM119* were comparable in all groups tested. All data are given as mean ± SD. ***P < 0.001; **P < 0.01. Scale bar: A-H = 33 μm

As GPNMB immunoreactivity was mainly present in brain areas known for robust amyloid deposition in 5XFAD mice, co-immunofluorescence stainings for GPNMB, IBA1 and Aβ were performed in 12-month-old animals in order to study spatial co-localization. Triple-labeling with GPNMB (red), IBA1 (green) and pan-Aβ (magenta) demonstrated that GPNMB protein was mainly detectable around amyloid plaque cores (Fig. 4a-d). In order to further investigate whether elevated GPNMB expression is a common phenomenon in AD transgenic mice, 12-month-old APP23 were analyzed using immunohistochemistry. Although abundant IBA1-positive microglia surrounded extracellular Aβ deposits in both 5XFAD and APP23 mice, GPNMB-immunoreactivity was restricted to the 5XFAD model, where it clustered around the central dense plaque core (Additional file 6) with microglia being consistently negative in 12-month-old APP23 mice (Fig. 4e-h and Additional file 4).

### GPNMB expression correlates with markers for disease-associated microglia

We further analyzed markers that have been proposed to be indicative of a subgroup of microglia cells under disease conditions, so called disease-associated microglia (DAMs) or microglial neurodegenerative phenotype (MGnD), but are absent or scarcely expressed in healthy animals [22, 24]. Indeed, levels of genes such as *CST7*, *TREM2*, *APOE*, *CLEC7a* or *CCL2* were found significantly up-regulated in 12-month-old 5XFAD mice compared to both WT and APP23 mice, while levels of homeostatic microglia genes like *AIF1* or *TMEM119* were unchanged (Fig. 4i). Significant correlations between *GPNMB* and *CST7*, *AIF1*, *TREM2*, *APOE*, *CLEC7a* and *CCL2* were observed while no correlation could be detected between *GPNMB* and the homeostatic microglia marker *TMEM119* (Additional file 7).

**Fig. 5:**
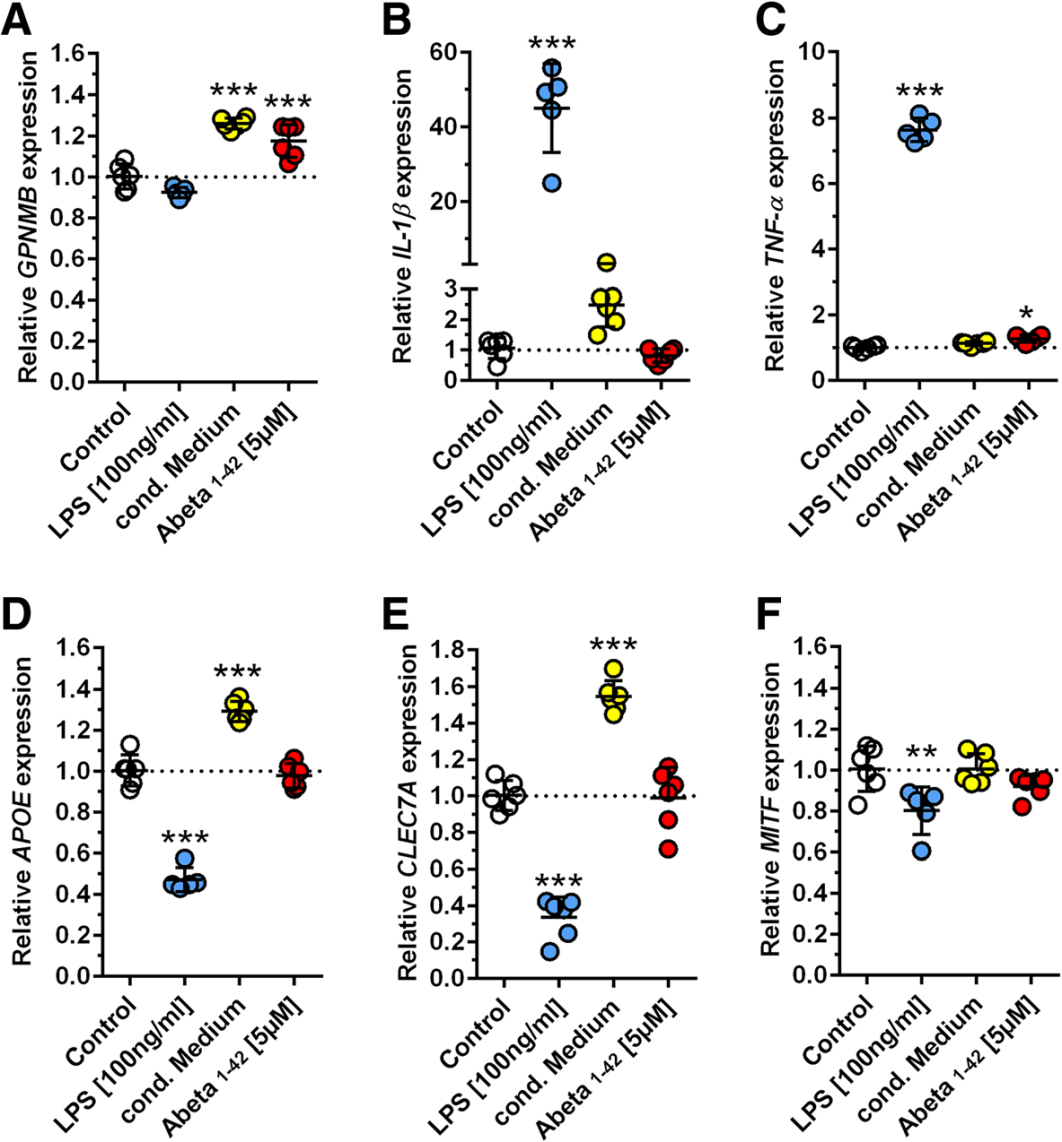
Soluble Aβ induces *GPNMB* mRNA expression in immortalized microglia cells. (**a**) Treatment of BV2 cells with Aβ-containing medium or synthetic Aβ_1–42_ peptides resulted in a highly significant increase in *GPNMB* mRNA expression, while LPS treatment had no effect on *GPNMB* levels. In contrast, LPS treatment caused microglia activation as shown by upregulation of the pro-inflammatory cytokines *IL1-β* and *TNFα* (**b**, **c**). While LPS treatment led to decreased expression of *APOE* and *CLEC7A*, conditioned Aβ-containing media induced these DAM phenotype-associated genes (**d**, **e**). *MITF* levels did not change following treatment with Aβ-conditioned medium or synthetic Aβ although *GPNMB* levels were clearly elevated (**f**). All data are given as mean ± SD. * *P* < 0.05; **P < 0.01; ***P < 0.001

Next, we assessed whether Aβ peptides were able to trigger *GPNMB* expression in vitro. To this end, the immortalized murine microglial cell line BV-2 was treated with 5 μM synthetic Aβ_1–42_ or conditioned medium derived from SH-SY5Y cells overexpressing human APP695 with the Swedish mutation. This medium was harvested after 48 h and contained mainly Aβ_1–40_ and Aβ_1–42_ (Additional file 8). Treatment with LPS was employed as a control condition to trigger an inflammatory reaction. Quantification of mRNA expression levels revealed a significant up-regulation of *GPNMB* in cells treated with Aβ_1–42_ or Aβ-conditioned medium while LPS treatment did not change *GPNMB* expression (Fig. 5a). Instead, LPS treatment led to a typical microglia activation pattern as indicated by up-regulation of genes encoding for pro-inflammatory cytokines such as *IL-1β* and *TNF* (Fig. 5b-c). *CLEC7A* and *APOE* representing DAM markers showed a significantly increased expression only after treatment with conditioned medium containing Aβ peptides (Fig. 5d-e). Surprisingly, the expression levels of the transcription factor MITF, which has been reported as an important regulator of *GPNMB* [9], was unchanged in conditions with elevated *GPNMB* expression (Fig. 5f).

### GPNMB in sporadic Alzheimer’s disease cases

**Fig. 6:**
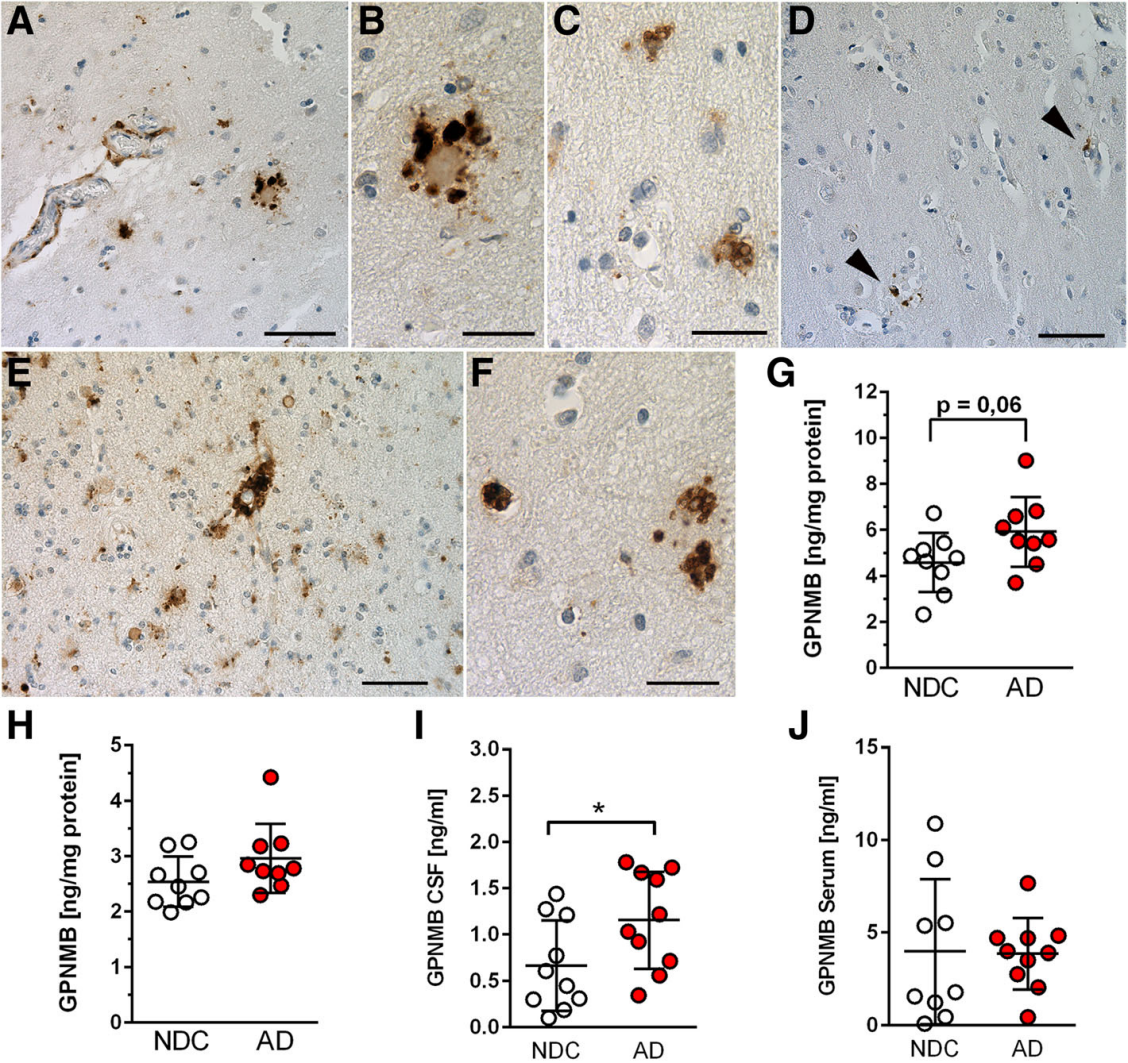
Increased GPNMB protein levels in brain tissue and cerebrospinal fluid (CSF) of sporadic Alzheimer‘s disease cases. GPNMB immunoreactivity was detected in microglial cells surrounding amyloid plaque cores and in the vicinity of blood vessel walls (**a**, **e**). (**b**) High-power view of the plaque core in (**a**). In addition, GPNMB-positive amoeboid microglia were detected in plaque-free areas (**c, f**), while GPNMB-positive microglia were only occasionally observed in samples from non-demented control patients (**d**). (**g**) In the TBS-soluble brain fractions, higher levels of GPNMB were detected in AD cases when compared to non-demented controls (NDC), however, without reaching statistical significance (*p* = 0.06). (**h**) No differences were detected between the two groups in SDS-soluble brain fractions. (**i**) The amount of GPNMB in the CSF of AD patients was significantly higher than in control patients. (**j**) No significant difference in GPNMB serum levels could be detected between controls and AD. All data are given as mean ± SD. *P < 0.05. Scale bar: A,D,E = 50 μm; B,C,F = 20 μm

Finally, we investigated whether our findings in mouse models of AD can be translated to human AD patients. Thus, brain samples from AD and NDC subjects were stained with a GPNMB antibody. The specificity of the antibody used for immunohistochemical staining of human brain tissues was verified with a blocking peptide which entirely abolished GPNMB immunoreactivity (Additional file 9). Interestingly, abundant GPNMB immunoreactivity was observed throughout cortical tissue samples from sporadic AD patients. In particular, intense GPNMB staining was detected in vessel walls and around amyloid plaque cores, confirming our observations in AD mouse models (Fig. 6a, b, e). However, also non-plaque-associated GPNMB-positive cells were frequently detected showing an amoeboid phenotype reminiscent of lipid-laden microglia in tissue samples from human patients (Fig. 6c, f). In non-demented controls, considerably less GPNMB immunoreactivity was observed and only occasionally GPNMB-positive cells were detected throughout the cortex (Fig. 6d).

We next measured TBS- and SDS-soluble GPNMB protein levels in lysates of the medial frontal gyrus of human patients with sporadic AD and non-demented controls. Quantification using a GPNMB-specific sandwich ELISA revealed that TBS-soluble GPNMB protein levels tended to be increased in AD patients in comparison to non-demented control individuals, but the difference did not reach statistical significance (*p* = 0.06; Fig. 6g). In the SDS-soluble fraction, no difference in GPNMB protein levels was noted between the two groups (Fig. 6h).

In addition, GPNMB protein levels were measured in the CSF and sera of a distinct cohort of patients suffering from sporadic AD as well as in non-demented controls. Importantly, GPNMB protein levels were found to be significantly increased in the CSF of sporadic AD patients when compared to non-demented controls (*p* < 0.05). (Fig. 6i). No differences in GPNMB levels between non-demented controls and sporadic AD patients were found in serum samples (Fig. 6j). In order to validate the GPNMB ELISA measurements presented in the current study intra-assay coefficients of variation (relative standard deviations) between duplicate reads (technical replicates on the same assay plate) were calculated for the different sample matrices and are summarized in Additional file 10. In addition, TBS- and SDS-soluble brain extracts from WT mice (*n* = 11) were measured on 2 different days. The mean inter-assay coefficients of variation were 4.7% (median 3.4%, range 0.8–19.9%) for TBS- and 15.7% (median 16.8%, range 8.9–20.3%) for SDS-soluble fractions.

## Discussion

Since therapeutic approaches against long established AD targets such as amyloid plaques and NFTs have so far not been successful, more recent therapeutic strategies try to tackle alternative targets, among which neuroinflammation is one of the most promising. Accumulating evidence suggests a critical role for microglia in the pathogenesis of AD, and latest human genetics data have identified several novel AD risk genes such as CD33 or CR1, which are highly expressed by these brain resident immune cells (reviewed in [21]). Two main hypotheses have been put forward regarding the role of reactive microglia in brain diseases. One is claiming that microglia is protective against CNS insults such as aggregated β-amyloid by promoting their clearance through phagocytosis. On the other hand, many findings indicate that chronic activation of microglia is harmful to neurons and contributes to disease progression and severity. In AD, while the detrimental effects of microglia activation seem to manifest in later stages of the disease, protective microglial activities supposedly occur in the early disease stages [10]. In order to develop therapeutic approaches that target microglia and modulate their behaviour, a better understanding of the proteins and molecular mechanisms involved in their activation and potential dysfunction in AD brains is required.

In our recent transcriptome analysis of the APP/PS1KI transgenic AD mouse model, which develops severe neurodegeneration, a variety of genes implicated in the neuroinflammatory response were identified as overexpressed [51]. One of the most strongly up-regulated genes was *GPNMB*, a transmembrane type I protein also known as osteoactivin. We here describe GPNMB as a novel AD-associated marker in both transgenic AD models and sporadic AD patients. Using immunohistochemical analyses, RT-PCR experiments and ELISA measurements, we were able to show that *GPNMB* is overexpressed in the APP/PS1KI and 5XFAD transgenic mouse models of AD in an age-dependent manner, with age-matched WT animals being consistently negative. Double-immunofluorescent staining using GPNMB and the microglia/macrophage marker IBA1 revealed a distinct co-localization, corroborating previous results in the inflamed rat brain, where GPNMB was found to co-localize with the microglia/macrophage marker OX42 [15]. Co-stainings with markers against GFAP to detect astrocytes or NeuN to detect neurons were consistently negative, underscoring the restricted microglial localization. This result is also in good agreement with recent data from a RNA-sequencing study, which demonstrated GPNMB expression primarily in microglia and, to a lesser extent, in oligodendrocyte precursor cells [60]. It is further supported by a recent transcriptome study demonstrating an upregulation of GPNMB in major histocompatibility complex (MHC) II-positive microglial cells isolated from the 5XFAD mouse model [58].

Employing multi-fluorescent staining using antibodies against GPNMB, IBA1 and Aβ, we found that GPNMB-positive microglia were primarily located in the close vicinity of extracellular plaques in the 5XFAD mouse model. In contrast, no GPNMB-immunoreactivity could be detected in APP23 mice, although this model also showed abundant extracellular plaque pathology surrounded by numerous IBA1-positive microglia cells [45]. This is also reflected in an earlier RNA microarray analysis of APP23 mice, in which no overexpression of GPNMB was reported [49]. In contrast, *GPNMB* was found to be up-regulated in a longitudinal gene profiling analysis of the 5XFAD mouse model [27], as well as in a transcriptome study investigating gene expression changes in CD11c-positive microglia isolated from the amyloid-depositing APPswe/PS1dE9 mouse model [20, 35]. While 5XFAD mice show cortical neuron loss at 12 months of age [7, 19], even higher cortical neuron numbers have been reported in 8-month-old APP23 compared to WT mice, which decrease in 27-month-old APP23 mice back to WT levels [2]. In addition, the amyloid plaque composition in APP23 mice is much different compared to human AD with Aβ_1–40_ representing the main component in APP23 while Aβ_1–42_ is the predominant species in human AD brain [42].

Activated microglia are characteristic for numerous neurodegenerative diseases aside from AD [50]. Studies integrating microglial/myeloid expression data sets from diverse neurodegenerative disease models, including AD transgenic mice, multiple sclerosis and ALS models, have suggested the presence of a particular microglia activation state, which has been termed either “microglial neurodegenerative phenotype” (MGnD) [24] or “disease-associated microglia” (DAM) [22]. Together with other genes (e.g. *CLEC7A*, *CCL2* or *FABP5*), *GPNMB* was found to be up-regulated in the MGnD profile. In addition, it has been proposed that Trem2 and ApoE are intimately linked to a switch from a homeostatic to a neurodegenerative microglial phenotype [24]. Based on these findings, we tested whether other MGnD genes might be induced in 5XFAD mice with elevated GPNMB levels, but not in APP23 mice, in which GPNMB expression was not increased. Indeed, the strong transcriptional activation of *GPNMB* and *CST7* in 12-month-old 5XFAD but not in APP23 mice, together with the unchanged expression levels of the microglia homeostatic genes *AIF1* and *TMEM119* was consistent with the idea that *GPNMB* might be regulated as part of the MGnD response. The exact role of the MGnD activation state is not clear. However, it has been shown that the injection of apoptotic neurons into the cortex and hippocampus of adult mice resulted in the induction of *APOE* and the up-regulation of other genes implicated in the MGnD profile, including *GPNMB* [24]. *APOE* expression was also up-regulated in our 5XFAD but not in the APP23 mice, together with additional MGnD-associated markers such as *CLEC7A* or *CCL2*. This could mean that the MGnD activation state is only triggered in the presence of dead or dying neurons, and fits well to the observation of abundant GPNMB-immunoreactivity in 5XFAD mice. In contrast to APP23 mice, which show no neocortical neuron loss even at 27 months of age [2], significantly reduced neuron numbers have been reported in deep cortical layers of 5XFAD mice beginning at nine months of age [7, 19, 54]. Similarly, APP/PS1KI mice also show a strong age-dependent induction of *GPNMB* expression, together with the up-regulation of several MGnD-associated genes such as *CLEC7A*, *ITGAX* or *CSF1* [51] and with robust hippocampal and cortical neuron loss [3, 6].

Additionally, we provide in vitro evidence that soluble Aβ might promote the switch in microglia gene expression from a “homeostatic” to a “disease-associated” state. Upon treatment of an immortalized microglial cell line with synthetic Aβ_1–42_ or Aβ-containing conditioned media, the expression levels of *GPNMB* as well as other MGnD-associated markers such as *APOE* and *CLEC7A* were highly increased. This indicates that soluble Aβ peptides are also capable of inducing GPNMB expression, in addition to aggregated Aβ as found in brain tissues from human AD or transgenic AD mice. In contrast, treatment of microglia cells with LPS induced a typical pro-inflammatory gene expression profile, with *GPNMB* levels being unchanged. Surprisingly, expression levels of the transcription factor MITF, which has been reported to be a critical regulator of GPNMB expression [9], was found to be down-regulated following LPS treatment. However, it has also been shown that LPS is capable of suppressing gene expression in macrophages by down-regulating factors such as MITF [18].

**Fig. 7:**
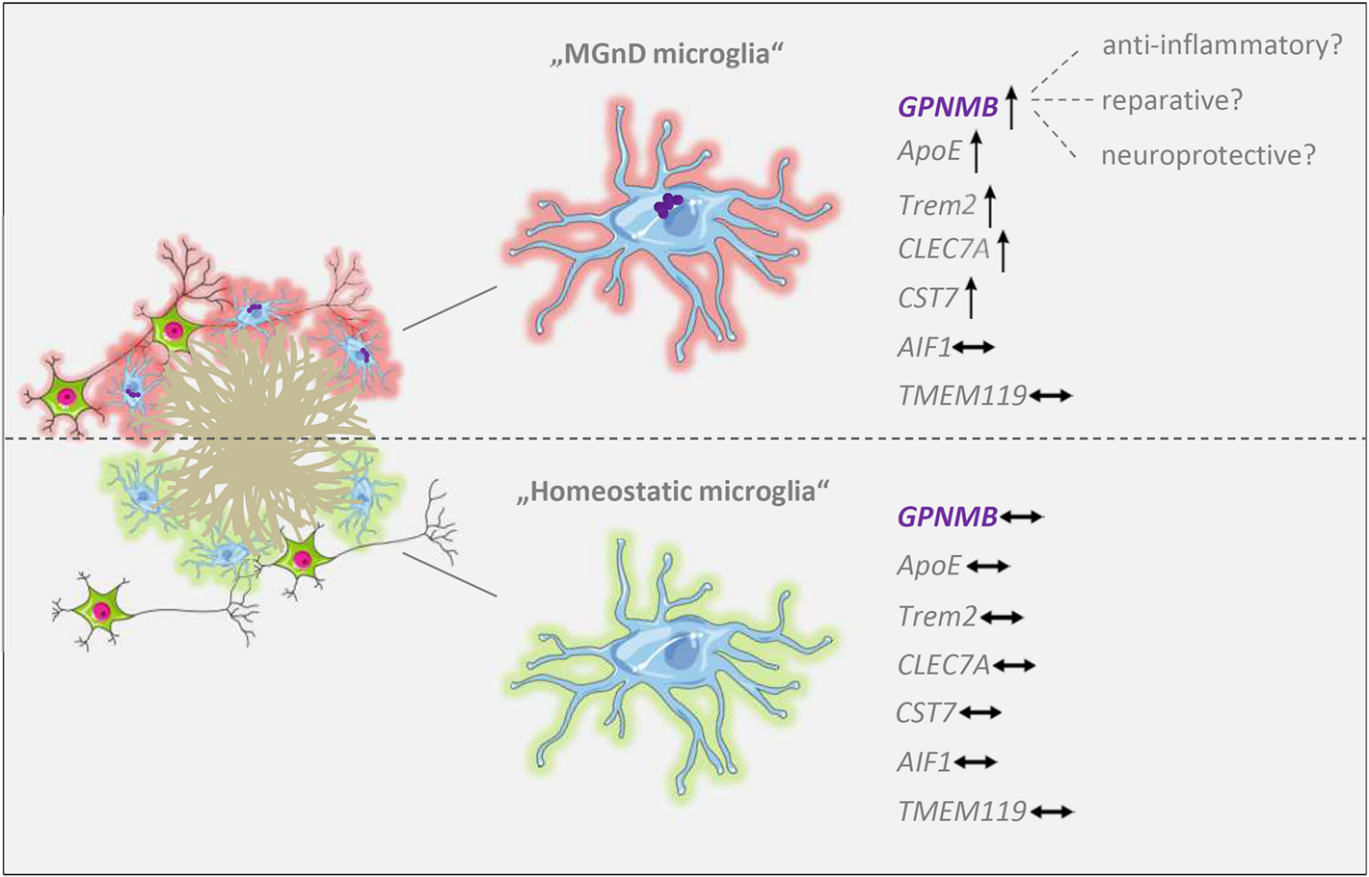
Schematic summary of results and hypothesis: In Alzheimer’s disease (AD) mouse models with profound neuron loss (e.g. 5XFAD), GPNMB-positive activated microglia cells cluster around individual amyloid plaque cores. In contrast, microglia cells are persistently GPNMB-negative in healthy wild-type mice or AD models showing abundant plaque pathology without neuron loss. Under neurodegenerative conditions, microglia switch from a homeostatic to a disease-associated phenotype, which has been called “microglial neurodegenerative phenotype” (MGnD) or “disease-associated microglia” (DAMs), and which is characterized by the upregulation of a subset of genes including GPNMB. The precise role of GPNMB in the central nervous system is not yet known, however, evidence indicates that it has anti-inflammatory and regenerative functions. Individual components of the scheme have been taken from: http://smart.servier.com

We suggest that Aβ itself could be partially responsible for the phenotypic switch of microglia to a neurodegenerative state during AD progression, although it is clearly not sufficient in vivo as shown by the lack of MGnD markers in APP23 mice. In conclusion, our findings in transgenic AD mouse models support that GPNMB is part of a microglial activation state that occurs in advanced disease stages and only in AD models showing profound cerebral neuron loss. Besides elevated GPNMB levels, this microglial activation state under neurodegenerative conditions is characterized by the upregulation of a subset of genes including *APOE*, *TREM2*, *CLEC7A* and *CST7*. Whether GPNMB has a protective or detrimental role in this context has to be elucidated, but available in vitro evidence would argue for an anti-inflammatory, regenerative role of GPNMB [32, 39, 59, 61] (Fig. 7).

Importantly, we also found GPNMB to be elevated in both brain tissue and CSF samples of sporadic AD patients. To the best of our knowledge, the current study is the first to report elevated GPNMB levels in human AD subjects. At present, due to the small group sizes, the current results have to be interpreted with caution. Further studies with larger cohorts will be required to confirm these observations. Nevertheless, our findings are partially similar to studies of TREM2, which has been proposed as a potential microglia-associated biomarker for AD progression and therapy monitoring. TREM2 is also a type-I transmembrane protein localized to the cell surface that can undergo ectodomain shedding by ADAM proteases, and soluble TREM2 (sTREM2) has been detected in body fluids including the CSF. Although not consistent between all published studies, elevated sTREM2 levels have been reported in CSF samples of sporadic AD patients versus non-demented controls [11, 12, 36, 47]. Taken together, these studies provide proof of concept evidence that a microglia-derived protein that is detectable in body fluids like TREM2 or GPNMB could be used as a biomarker to monitor disease onset and progression, and might even function as a prognostic marker.

Elevated GPNMB levels were also reported in several other neurodegenerative disorders apart from AD. In patients with sporadic ALS, a disease characterized by the degeneration of motor neurons in the cerebral cortex and spinal cord, extracellular GPNMB aggregates were found in the grey and white matter of spinal cord tissue [30]. Consistent with this finding, the same group reported increased levels of GPNMB in the CSF and serum of ALS patients [48]. Elevated levels of GPNMB have also been shown in brains, CSF and plasma of Gaucher disease patients [23, 62]. Furthermore, the glycoprotein has been reported to be increased in Niemann-Pick Type C disease as well as in Tay-Sachs- and Sandhoff disease [28]. These diseases belong to the group of lysosomal storage disorders (LSDs), which are characterized by the abnormal accumulation of cellular debris in lysosomes and subsequent neurodegeneration [37]. Interestingly, a two-stage genome-wide association (GWA) meta-analysis associated GPNMB with a higher risk for Parkinson’s disease (PD) [17]. However, follow-up studies with independent cohorts of patients did not corroborate these initial findings [56, 57]. Given the involvement of GPNMB in other neurodegenerative diseases besides AD, it is unlikely to serve as a disease-specific biomarker. However, this does not exclude the possibility that multiplexing putative microglia-derived markers such as TREM2 and GPNMB with other proteins that might be discovered in future studies could result in disease-specific marker signatures. Apart from that, CSF GPNMB levels could potentially be used to monitor disease progression. In mice, we could demonstrate that GPNMB levels increased with age and disease progression. As the transgenic mouse models employed in the current study address in particular amyloid pathology, other models reflecting further pathological hallmarks have to be considered in future studies. In addition, longitudinal studies will be required to translate these findings into human patients and to prove that GPNMB levels correlate with the onset and course of human AD.

## Conclusions

Finally, as GPNMB has been shown to negatively regulate inflammatory responses [39], it is tempting to speculate that the protein is induced in neurodegenerative conditions such as AD or LSDs to restrain inflammation and to protect neurons. Therefore, increasing GPNMB levels in the CNS might be a potential future therapeutic strategy. However, more research to elucidate the precise role of GPNMB in the inflammatory responses associated with neurodegenerative diseases is clearly required.

## Additional files

Additional file 1: Characteristics of the study cohorts used for ELISA measurements. (PDF 233 kb)

Additional file 2: List of primers used for quantitative Real-Time PCR. (PDF 153 kb)

Additional file 3: Quantification of Aβ plaque load and GPNMB immunoreactivity in 5XFAD mice. (PDF 434 kb)

Additional file 4: Immunostaining against Aβ and GPNMB in parallel sections in the cortex of a 12-month-old APP23 mouse. (PDF 285 kb)

Additional file 5: Correlation analysis between GPNMB-ELISA (TBS- and SDS-soluble fractions) and GPNMB expression levels measured by RT-PCR (PDF 30 kb)

Additional file 6: High magnification of an amyloid plaque surrounded by GPNMB-positive microglia. (PDF 481 kb)

Additional file 7: Correlation analysis between GPNMB gene expression levels and microglia markers measured by RT-PCR. (PDF 121 kb)

Additional file 8: SDS-PAGE and Western-blot of Aβ peptides from the supernatant of APP695sw-transfected SH-SY5Y cells. (PDF 207 kb)

Additional file 9: Peptide competition assay to block GPNMB immunoreactivity in immunohistochemical stainings. (PDF 190 kb)

Additional file 10: Intra-assay variation of the ELISA measurements of the different sample matrices. (PDF 106 kb)
