# Supplementary material for: Glycoprotein NMB: a novel Alzheimer’s disease associated marker expressed in a subset of activated microglia
Source: Acta Neuropathol Commun. 2018 Oct 19;6:108. doi: 10.1186/s40478-018-0612-3 (PMC6194687; doi:10.1186/s40478-018-0612-3)
Supplement: Supplementary file 1 — Characteristics of the study cohorts used for ELISA measurements. (PDF 233 kb) [file 40478_2018_612_MOESM1_ESM.pdf]

Additional file 1:

Characteristics of the study cohorts used for ELISA measurements

Demographic data and neuropathological data for the human donors as provided by the Netherlands Brain Bank (NBB) and correlation analysis for GPNMB levels in brain extracts and neuropathological measures.

	AD	NDC	*p*-value
Number	9	9	
Gender (M/F)	2/7	2/7	> 0.999
Age (y)	79.78 ± 11.28	82.00 ± 9.77	0.5884

Case	Sex	Age (y)	PMD	Braak stage	Amyloid
AD-1	M	78	07:45	V	C
AD-2	M	82	05:05	V	C
AD-3	F	82	04:20	VI	C
AD-4	F	54	06:35	VI	C
AD-5	F	85	05:10	VI	C
AD-6	F	86	05:40	VI	C
AD-7	F	96	05:50	V	C
AD-8	F	77	06:05	V	C
AD-9	F	78	03:45	VI	C
NDC-1	F	85	04:40	II	A
NDC-2	F	85	05:19	II	B
NDC-3	M	96	05:23	I	B
NDC-4	F	60	07:30	I	A
NDC-5	F	77	02:55	I	B
NDC-6	F	82	05:10	II	0
NDC-7	F	84	06:55	I	0
NDC-8	M	81	07:55	II	0
NDC-9	F	88	06:15	n.d.	B

n.d.: not determined

Correlation	Spearman r	95% confidence interval	P value
GPNMB-TBS (ng/mg) - Amyloid score	0.2848	-0.2243 to 0.6717	0.2521
GPNMB-TBS (ng/mg) - Braak stage	0.3251	-0.1993 to 0.7047	0.2014
GPNMB-SDS (ng/mg) - Amyloid score	0.3838	-0.1160 to 0.7285	0.1159
GPNMB-SDS (ng/mg) - Braak stage	0.605	-0.1605 to 0.7244	0.1547

Characteristics of the study cohort used for CSF and serum measurements.

	AD	NDC	*p*-value
Number	10	10	
Gender (M/F)	8/2	5/5	0.3498
Age (y)	70.4 ± 7.6	62.5 ± 9.3	0.052
CSF Aβ (pg/ml)	472.9 ± 106.2 (n = 10)	1637 ± 215.4(n = 4)	<0.0001
CSF tTau (pg/ml)	756.1 ± 298.1 (n = 10)	457.8 ± 300.8 (n =4)	0.1172
CSF pTau (pg/ml)	96.5 ± 60.97 (n = 10)	40.00 ± 41.46 (n = 4)	0.1181

Case	Sex	Age (y) at LP
AD-10	F	59
AD-11	F	68
AD-12	F	76
AD-13	F	73
AD-14	M	61
AD-15	F	66
AD-16	F	82
AD-17	F	75
AD-18	M	78
AD-19	F	66
NDC-10	F	76
NDC-11	M	74
NDC-12	M	74
NDC-13	F	63
NDC-14	F	64
NDC-15	F	51
NDC-16	M	57
NDC-17	M	57
NDC-18	F	57
NDC-19	M	52
