# Supplementary material for: Glycoprotein NMB: a novel Alzheimer’s disease associated marker expressed in a subset of activated microglia
Source: Acta Neuropathol Commun. 2018 Oct 19;6:108. doi: 10.1186/s40478-018-0612-3 (PMC6194687; doi:10.1186/s40478-018-0612-3)
Supplement: Supplementary file 2 — List of primers used for quantitative Real-Time PCR. (PDF 153 kb) [file 40478_2018_612_MOESM2_ESM.pdf]

Additional file 2:

Primers used for RT-PCR in the present study:

**Gene**	**Forward (5' – 3')**	**Reverse (5' – 3')**
β-ACTIN	ATGGAGGGGAATACAGCCC	TTCTTTGCAGCTCCTTCGTT
AIF1 (IBA1)	CAGACTGCCAGCCTAAGACA	AGGAATTGCTTGTTGATCCC
APOE	GAGCTGATCTGTCACCTCCG	GGACTTGTTTCGGAAGGAGC
CCL2	GGGATCATCTTGCTGGTGAA	AGGTCCCTGTCATGCTTCTG
CLEC7a	AAGCTTTCCTGGGGAACTGT	CCAAACATCGTCTCACCGTA
CST7	AAGCACTCCTGGGTTATTGG	TGCCTAACTTCTGACACCCA
TMEM119	ATAGCTCAACATGGTCCCCTGGTTC	GGCCTGTTAGACACTGGGGGAGAC
TREM2	CCCTCGAAACTCGATGACTC	TGCAGAAAGTACTGGTGGAGG
MITF	TTGATGGATCCGGCCTTGCAAATG	TATGTTGGGAAGGTTGGCTGGACA

Primers for murine TGFβ1 and IL1-β were purchased from Qiagen as verified primer sets (QuantiTect Primer Assays).
