# Supplementary material for: Glycoprotein NMB: a novel Alzheimer’s disease associated marker expressed in a subset of activated microglia
Source: Acta Neuropathol Commun. 2018 Oct 19;6:108. doi: 10.1186/s40478-018-0612-3 (PMC6194687; doi:10.1186/s40478-018-0612-3)
Supplement: Supplementary file 3 — Quantification of Aβ plaque load and GPNMB immunoreactivity in 5XFAD mice. (PDF 434 kb) [file 40478_2018_612_MOESM3_ESM.pdf]

Additional File 3:

<u>Quantification of Aβ plaque load and GPNMB immunoreactivity</u>

Aβ plaque load and GPNMB immunoreactivity were quantified using 2.5-, 7- and 12-month-old 5XFAD mice (*n* = 5 per time point). In brief, three paraffin sections per animal, which were at least 30 µm distant from each other, were stained simultaneously with DAB. The relative Aβ plaque load and GPNMB signals in cortex, subiculum, thalamus and dentate gyrus were evaluated using a BX51 microscope (Olympus, Center Valley, PA, USA) equipped with a Moticam Pro 282 camera (Motic, Wetzlar, Germany) and the ImageJ software package (V1.41, NIH, USA). For each section, representative images from respective brain areas were captured, binarized to 8-bit black-and-white images using a fixed intensity threshold and the percentage covered by DAB was measured.

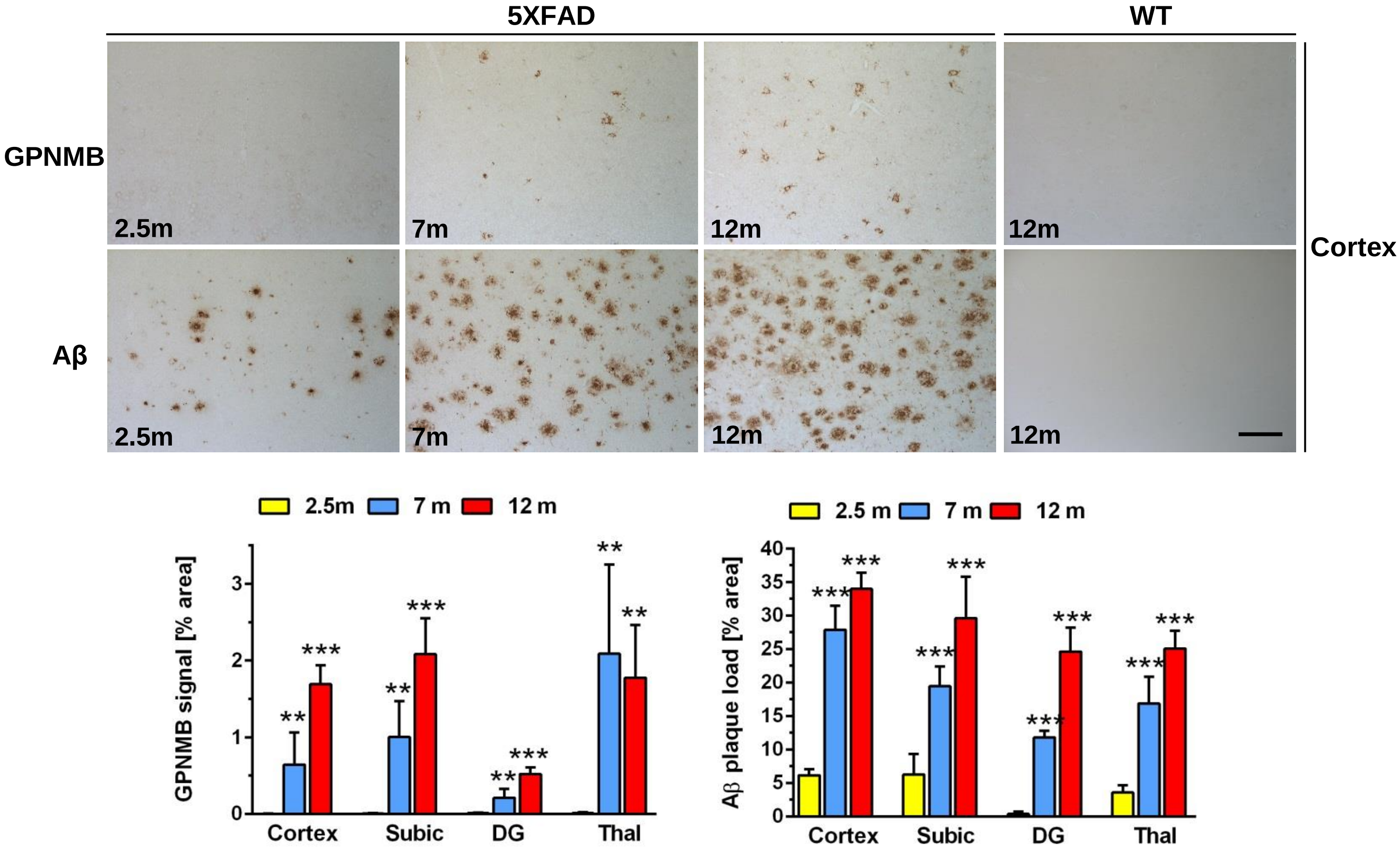

GPNMB expression increases in parallel with amyloid deposition in 5XFAD mice. (A) Representative pictures of cortical GPNMB- and Aβ immunoreactivity in 2.5-, 7- and 12-month-old 5XFAD mice and 12-month-old WT mice as a negative control. Quantification of GPNMB immunoreactivity (**A**) and extracellular Aβ plaque load (**B**) in 2.5-, 7- and 12-month-old 5XFAD mice in cortex, subiculum (Subic), dentate gyrus (DG) and thalamus (Thal). All data were given as mean ± standard deviation (SD). ***P<0.001; **P<0.01. Scale bar = 50 µm.
