# Supplementary material for: Glycoprotein NMB: a novel Alzheimer’s disease associated marker expressed in a subset of activated microglia
Source: Acta Neuropathol Commun. 2018 Oct 19;6:108. doi: 10.1186/s40478-018-0612-3 (PMC6194687; doi:10.1186/s40478-018-0612-3)

Additional file 4:

Immunostaining against Aβ and GPNMB in parallel sections in the cortex of a 12-month-old APP23 mouse. Inset shows higher magnification of the boxed area. Scale bar: 200 µm

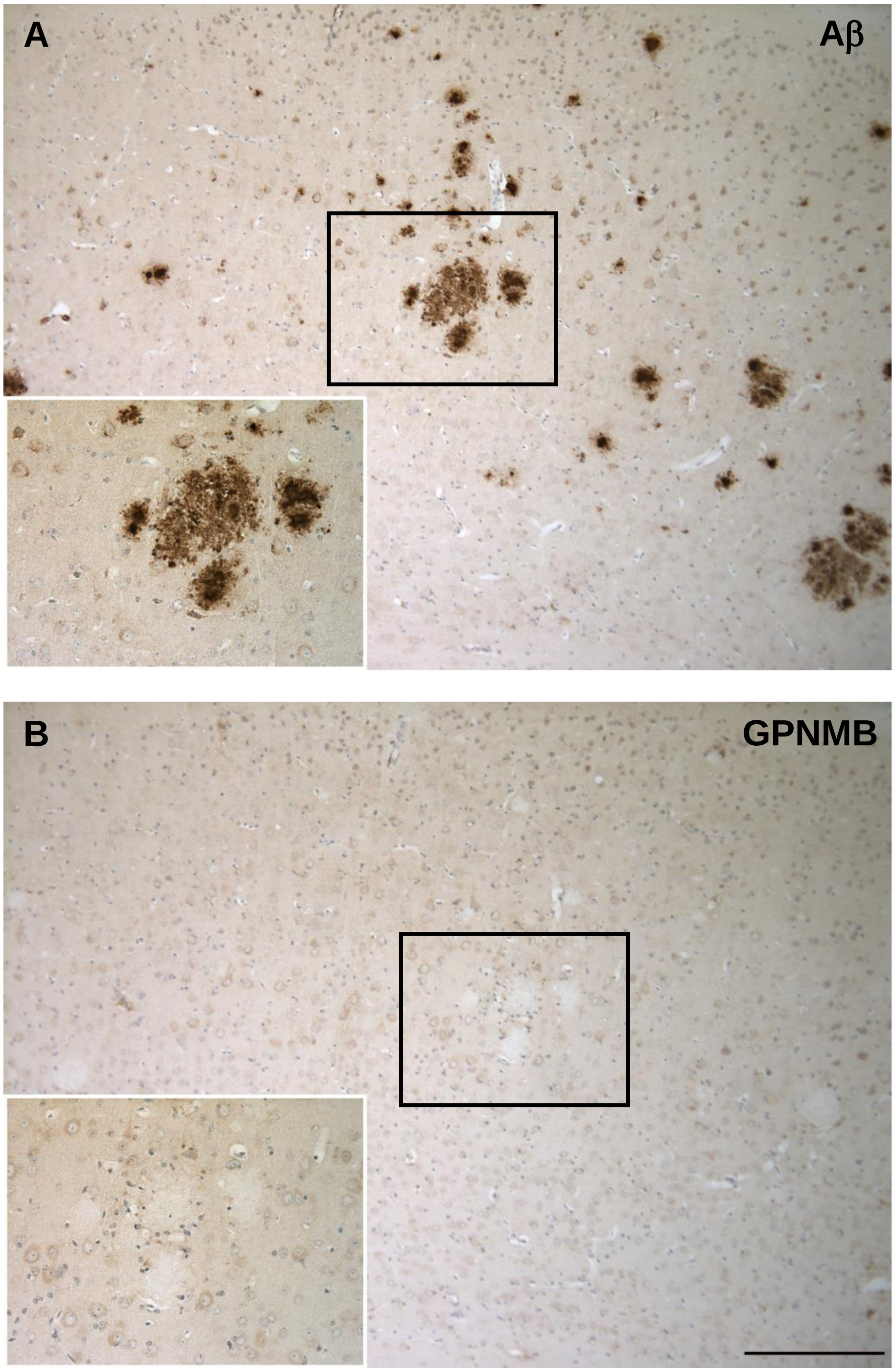

Supplement: Supplementary file 4 — Immunostaining against Aβ and GPNMB in parallel sections in the cortex of a 12-month-old APP23 mouse. (PDF 285 kb) [file 40478_2018_612_MOESM4_ESM.pdf]
