# Supplementary material for: Glycoprotein NMB: a novel Alzheimer’s disease associated marker expressed in a subset of activated microglia
Source: Acta Neuropathol Commun. 2018 Oct 19;6:108. doi: 10.1186/s40478-018-0612-3 (PMC6194687; doi:10.1186/s40478-018-0612-3)

Additional file 5:

Correlation analysis between GPNMB-ELISA (TBS- and SDS-soluble fractions) and GPNMB expression levels measured by RT-PCR in a subset of mice were both protein and RNA samples were available from the same animal (n = 14).

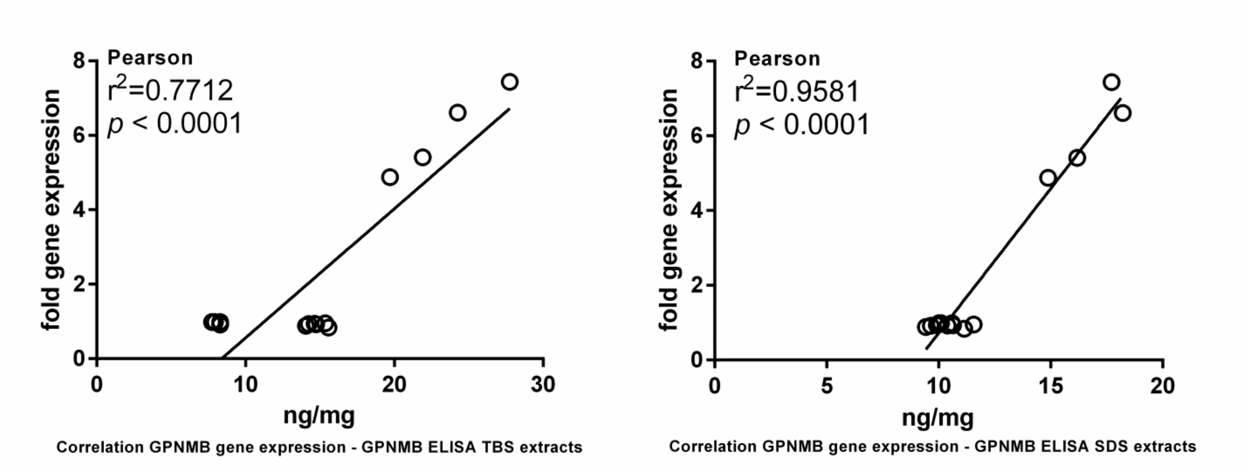

Supplement: Supplementary file 5 — Correlation analysis between GPNMB-ELISA (TBS- and SDS-soluble fractions) and GPNMB expression levels measured by RT-PCR (PDF 30 kb) [file 40478_2018_612_MOESM5_ESM.pdf]
