# Supplementary material for: Glycoprotein NMB: a novel Alzheimer’s disease associated marker expressed in a subset of activated microglia
Source: Acta Neuropathol Commun. 2018 Oct 19;6:108. doi: 10.1186/s40478-018-0612-3 (PMC6194687; doi:10.1186/s40478-018-0612-3)

Additional file 6:

<u>High magnification of an amyloid plaque surrounded by GPNMB-positive microglia</u>

(**A**) Aβ (green, antibody IC16) and GPNMB (red) staining in the cortex of a 12-month-old 5XFAD mouse revealed GPNMB-positive microglia clustering around a central dense plaque core without labeling the plaque periphery (dashed line).
(**B**) Higher magnification of the area indicated in A.

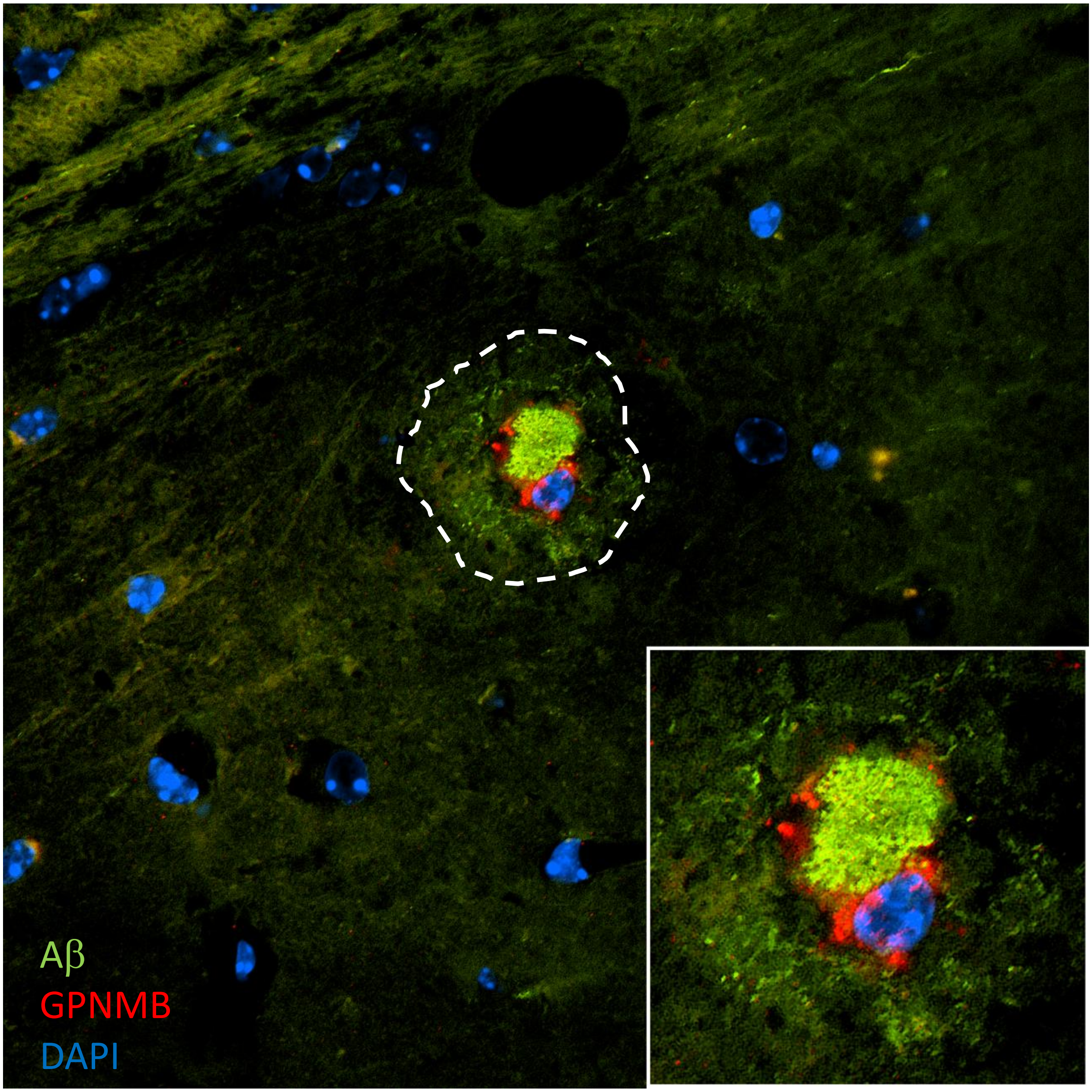

Supplement: Supplementary file 6 — High magnification of an amyloid plaque surrounded by GPNMB-positive microglia. (PDF 481 kb) [file 40478_2018_612_MOESM6_ESM.pdf]
