# Supplementary material for: Glycoprotein NMB: a novel Alzheimer’s disease associated marker expressed in a subset of activated microglia
Source: Acta Neuropathol Commun. 2018 Oct 19;6:108. doi: 10.1186/s40478-018-0612-3 (PMC6194687; doi:10.1186/s40478-018-0612-3)

**Additional file 7:**

<u>Correlation analysis between GPNMB gene expression levels and microglia markers measured by RT-PCR.</u>

Significant correlations between *GPNMB* and *CST7*, *AIF1*, *TREM2*, *APOE*, *CLEC7a* and *CCL2* were observed while no correlation could be detected between *GPNMB* and the homeostatic microglia marker *TMEM119*.

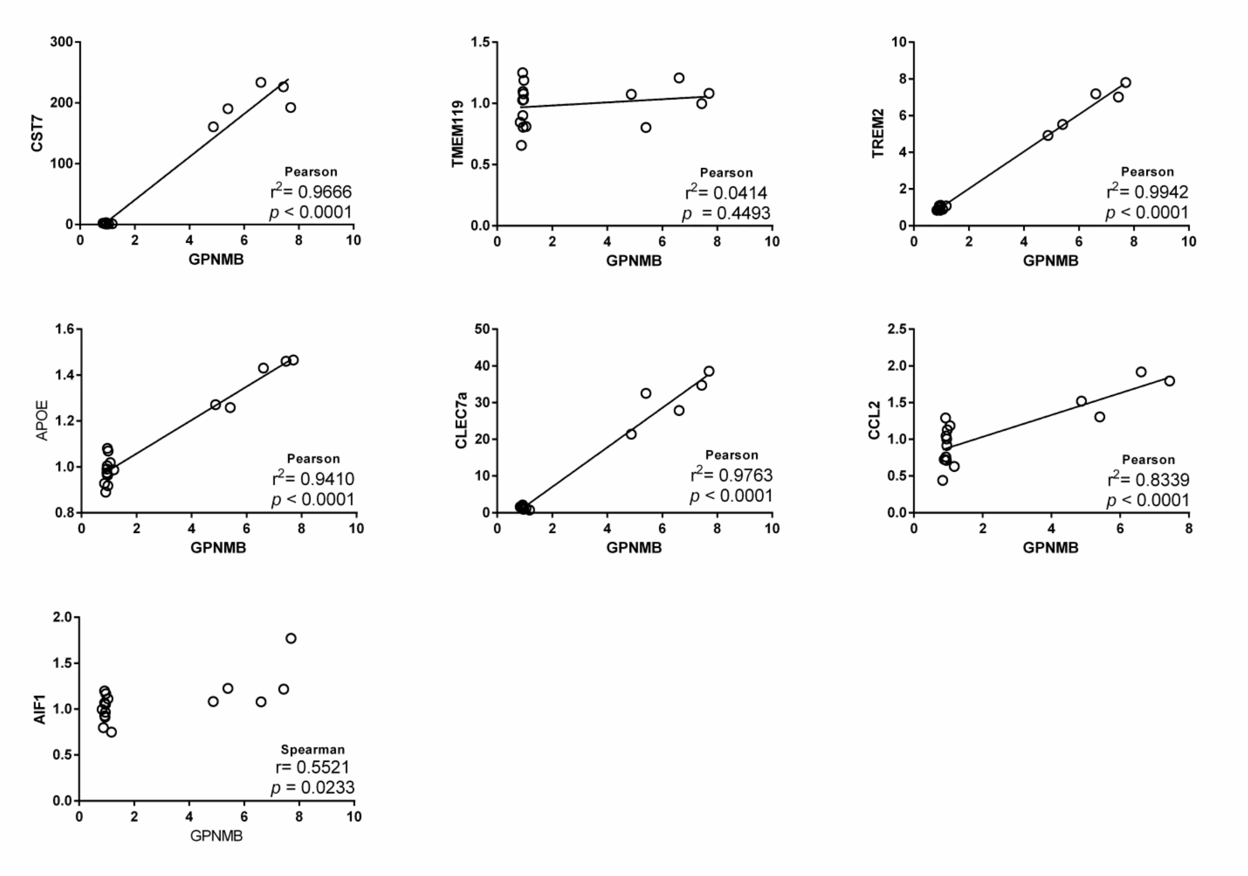

Supplement: Supplementary file 7 — Correlation analysis between GPNMB gene expression levels and microglia markers measured by RT-PCR. (PDF 121 kb) [file 40478_2018_612_MOESM7_ESM.pdf]
