# Supplementary material for: Glycoprotein NMB: a novel Alzheimer’s disease associated marker expressed in a subset of activated microglia
Source: Acta Neuropathol Commun. 2018 Oct 19;6:108. doi: 10.1186/s40478-018-0612-3 (PMC6194687; doi:10.1186/s40478-018-0612-3)
Supplement: Supplementary file 8 — SDS-PAGE and Western-blot of Aβ peptides from the supernatant of APP695sw-transfected SH-SY5Y cells. (PDF 207 kb) [file 40478_2018_612_MOESM8_ESM.pdf]

Additional file 8:

SDS-PAGE and Western Blot

A mix of synthetic Aβ-peptides (Aβ$_{1-37}$, Aβ$_{1-38}$, Aβ$_{1-39}$, Aβ$_{1-40}$ and Aβ$_{1-42}$) (AnaSpec) as well as cell culture supernatant of SH-SY5Y APP695 cells containing secreted Aβ-peptides was separated on 10% T/5% C polyacrylamide gels by urea bicine/tris SDS-PAGE (Klafki, 1996). The separated peptides where electrophoretically transferred onto PVDF-membranes (Biorad) at 0.75 mA/cm^2 under semi-dry conditions for 30 min. In order to improve antibody binding, the PVDF-membranes were then boiled in pre-warmed PBS for 3 min in a microwave. Afterwards, the membranes were blocked with 2% ECL prime blocking reagent (GE Healthcare) in PBS-T for 1h at room temperature. Immunostaining was performed with the anti-Aβ antibody 1E8 (Nanotools) overnight at 4°C. The next day, the membranes were washed three times in PBS-T before they were incubated with a biotinylated anti-mouse IgG (Linaris) for 1h at room temperature. Then, membranes were washed again in PBS-T before incubation with a streptavidin horseradish peroxidase complex in PBS-T (GE Healthcare). The membranes were developed with ECL-plus chemiluminescence (GE Healthcare) according to the manufacturer and the bands were visualized using a Fusion SL Imager (Vilber).

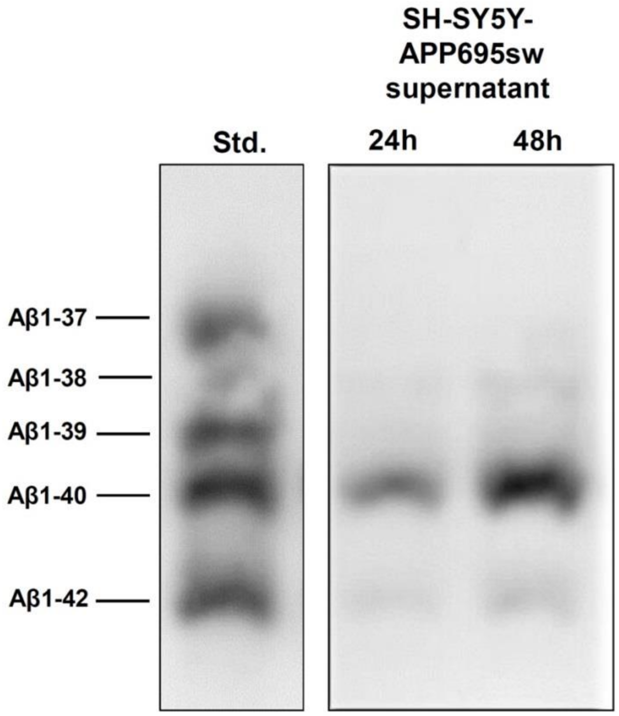

Figure S2: Medium from APP695sw-transfected SH-SY5Y cells contained mainly Aβ$_{1-40}$ and Aβ$_{1-42}$ after 48h.
