# Supplementary material for: Glycoprotein NMB: a novel Alzheimer’s disease associated marker expressed in a subset of activated microglia
Source: Acta Neuropathol Commun. 2018 Oct 19;6:108. doi: 10.1186/s40478-018-0612-3 (PMC6194687; doi:10.1186/s40478-018-0612-3)
Supplement: Supplementary file 9 — Peptide competition assay to block GPNMB immunoreactivity in immunohistochemical stainings. (PDF 190 kb) [file 40478_2018_612_MOESM9_ESM.pdf]

Additional file 9:

Peptide competition assay

In order to further investigate the specificity of the GPNMB antibody (sc-47006, Santa Cruz), an immunoadsorption experiment was carried out using the respective blocking peptide (sc-47006P). 1 µg GPNMB peptide was incubated with the antibody (1:200) in phosphate-buffered saline (PBS) for 5 h at room temperature with continuous agitation and followed by centrifugation at 14.000 x g for 5 min as published previously. The supernatant was used for immunohistochemistry as described in the Material and Methods part.

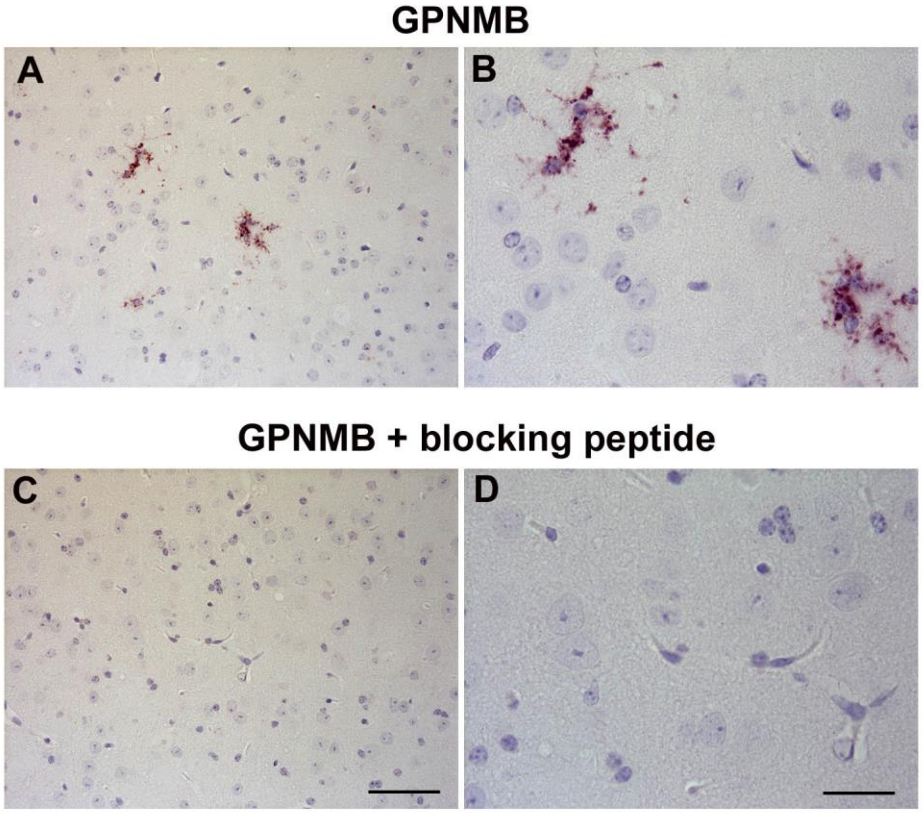

Figure S3: The use of 1 µg blocking peptide completely abolished GPNMB immunoreactivity in a human AD case. Scale bar: A,C = 50 µm; B,D = 20 µm
