# Supplementary material for: Glycoprotein NMB: a novel Alzheimer’s disease associated marker expressed in a subset of activated microglia
Source: Acta Neuropathol Commun. 2018 Oct 19;6:108. doi: 10.1186/s40478-018-0612-3 (PMC6194687; doi:10.1186/s40478-018-0612-3)
Supplement: Supplementary file 10 — Intra-assay variation of the ELISA measurements of the different sample matrices. (PDF 106 kb) [file 40478_2018_612_MOESM10_ESM.pdf]

Additional file 10:

Intra-assay variation of the ELISA measurements of the different sample matrices

Intra-assay variation of the ELISA measurements of the different sample matrices

Assay	Sample matrix	n	mean intra-assay CV*	median	range
Mouse Osteoactivin/ GPNMB ELISA	TBS brain extracts from 12 months old mice	40	3,02%	2,19%	0.11-16.01%
Mouse Osteoactivin/ GPNMB ELISA	SDS brain extracts from 12 months old mice	40	3,35%	2,76%	0.28-10.99%
Mouse Osteoactivin/ GPNMB ELISA	TBS spinal cord extract from 12 months old mice	8	1,28%	2,17%	0.25-3.05%
Mouse Osteoactivin/ GPNMB ELISA	SDS spinal cord extract from 12 months old mice	8	4,17%	4,03%	0.23-8.85%
Human Osteoactivin / GPNMB ELISA	Human cerebrospinal fluid	19	9,57%	8,70%	0.41-20.5%
Human Osteoactivin / GPNMB ELISA	Human blood serum	19	17,21%	5,91%	0.38-141.42%
Human Osteoactivin / GPNMB ELISA	Human brain TBS extracts	18	3,74%	2,86%	0.11-8.77%
Human Osteoactivin / GPNMB ELISA	Human brain SDS extracts	18	3,49%	2,61%	0-16.9%

* The GPNMB levels in all samples were measured in duplicates. For each individual sample the % CV (relative standard deviation) was calculated by dividing the SD of the duplicates by their mean and multiplying by 100. For each sample type the average of the individual CVs is reported.
